# Sexual Dimorphism Within Brain Regions Controlling Speech Production

**DOI:** 10.3389/fnins.2019.00795

**Published:** 2019-07-30

**Authors:** Laura de Lima Xavier, Sandra Hanekamp, Kristina Simonyan

**Affiliations:** ^1^Department of Otolaryngology Head and Neck Surgery, Massachusetts Eye and Ear Infirmary, Harvard Medical School, Boston, MA, United States; ^2^Department of Neurology, Massachusetts General Hospital, Harvard Medical School, Boston, MA, United States

**Keywords:** speech control, sensorimotor network, cortical thickness, fMRI, healthy subjects

## Abstract

Neural processing of speech production has been traditionally attributed to the left hemisphere. However, it remains unclear if there are structural bases for speech functional lateralization and if these may be partially explained by sexual dimorphism of cortical morphology. We used a combination of high-resolution MRI and speech-production functional MRI to examine cortical thickness of brain regions involved in speech control in healthy males and females. We identified greater cortical thickness of the left Heschl’s gyrus in females compared to males. Additionally, rightward asymmetry of the supramarginal gyrus and leftward asymmetry of the precentral gyrus were found within both male and female groups. Sexual dimorphism of the Heschl’s gyrus may underlie known differences in auditory processing for speech production between males and females, whereas findings of asymmetries within cortical areas involved in speech motor execution and planning may contribute to the hemispheric localization of functional activity and connectivity of these regions within the speech production network. Our findings highlight the importance of consideration of sex as a biological variable in studies on neural correlates of speech control.

## Introduction

Speech production is a complex motor behavior that requires the involvement of several brain regions and their respective networks, which collectively support different aspects of auditory and phonological processing, sensorimotor integration (SMG), executive function, motor planning and execution (Simonyan and Fuertinger, 2015). Contrary to the empirical notion of left-hemispheric lateralization of brain activity during speech production, several recent studies defined a bilateral functional and structural distribution of the large-scale speech network (Simonyan et al., 2009; Morillon et al., 2010; Gehrig et al., 2012; Silbert et al., 2014; Simonyan and Fuertinger, 2015; Kumar et al., 2016). Within this network, a hemispheric lateralization of functional activity and connectivity was found to be a feature of *selected* brain regions and their subnetworks. While these studies refined our understanding of the hemispheric lateralization of speech production, its potential physiological underpinnings remain poorly understood. A recent multimodal study combining functional MRI (fMRI), intracranial electroencephalographic (EEG) recordings and large-scale neural population simulations based on diffusion-weighted MRI has demonstrated a direct modulatory role of dopaminergic neurotransmission on a functional lateralization of nigro-striatal and nigro-motocortical pathways involved in speech production (Fuertinger et al., 2018). Given the previous reports of sex differences in perceptual aspects of speech and language neural representations (Binder et al., 1995; Frost et al., 1999; Kansaku and Kitazawa, 2001; Clements et al., 2006), it is plausible to assume that another factor contributing to cortical hemispheric lateralization during speech production may be rooted in sex-specific differences of structural brain organization. Along these lines, it has been suggested that females have a more bilateral language representation, while language processing is mostly left-lateralized in males (McGlone, 1980; Dorion et al., 2000; Gur et al., 2000). For example, males show left-hemispheric activation during phonological tasks, while females show largely bilateral activity (Shaywitz et al., 1995). Male stroke patients have been reported to exhibit verbal impairments more frequently after lesions of the left hemisphere than females (McGlone, 1980; Hier et al., 1994), although sex differences were not replicated in other stroke studies involving unilateral lesions (Basso, 1992; Pedersen et al., 1995, 2004). Several studies, including large meta-analyses, have also failed to identify sex-specific differences in brain lateralization (Binder et al., 1995; Frost et al., 1999; Kansaku and Kitazawa, 2001; Sommer et al., 2004; Kitazawa and Kansaku, 2005; Clements et al., 2006; Wallentin, 2009; Kong et al., 2018). However, it should be noted that these studies have primarily focused on perceptual and cognitive aspects of speech and language processing and have not specifically examined the motor aspects of speech control. Inconsistencies in findings might also stem from high functional heterogeneity that characterizes large atlas-based macroanatomic labels as used in previous studies. Therefore, to circumvent these limitations and to focus on the speech production system, we examined the presence of sex differences in cortical thickness (CT) in brain regions that are functionally active during real-life speech production in healthy males and females. We hypothesized that hemispheric lateralization of regional brain activity during speech production may, in part, be explained by sex-specific asymmetry in cortical morphology within the speech controlling network.

## Materials and Methods

### Study Subjects

A total of 109 subjects participated in the study, including 59 healthy females (mean age 50.4 ± 10.5 years) and 50 age-matched healthy males (mean age 51.9 ± 9.3 year). All subjects were monolingual native English speakers, right-handed as determined by the Edinburgh Handedness Inventory (Oldfield, 1971), had normal cognitive performance and lexical verbal fluency as determined by the Mini-Mental State Examination (Cummings, 1993), and had no history of speaking, hearing, psychiatric or neurological problems. There were no differences in mean age and the education level between the male and female groups (*p* > 0.46). This study was carried out in accordance with the recommendations of the Internal Review Board of Massachusetts Eye and Ear Infirmary. All subjects gave written informed consent in accordance with the Declaration of Helsinki.

### Image Acquisition

All subjects underwent high-resolution MRI on 3.0 T Philips scanner with an 8-channel Sense head coil. An anatomical scan was acquired in all subjects using a T1-weighted MPRAGE sequence (flip angle = 8°, TR = 7.5 ms, TE = 2 ms, FOV = 210 × 210 mm^2^, 172 slices with an isotropic voxel size of 1 mm^3^). Among these, 16 females (mean age 50.9 ± 9.6 years) and 13 age-matched males (mean age 52.3 ± 9.0 years) participated in an additional whole-brain fMRI scan using a gradient-weighted echo planar imaging (EPI) pulse sequence and blood oxygen level dependent (BOLD) contrast (TR = 10.6 s, including an 8.6 s delay for listening to and production of the task and 2 s for image acquisition, TE = 30 ms, flip angle = 90°, 36 contiguous slices, slice thickness = 4 mm, matrix size = 64 × 64 mm, FOV = 240 × 240 mm^2^). A sparse-sampling event-related fMRI design was used to minimize scanner noise, task-related acoustic interferences, and orofacial motion (Gracco et al., 2005; Blackman and Hall, 2011; Adank, 2012).

**FIGURE 1:**
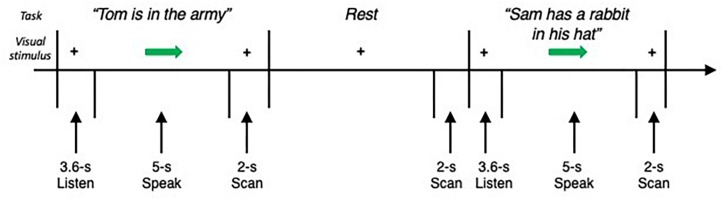
Schematic illustration of the experimental fMRI design. The subject fixated on the cross and listened to the acoustically presented sentence for a 3.6-s period. Sentences were pseudorandomized and presented one at a time. No stimulus was presented for the baseline resting condition, during which the subject fixated on the cross. An arrow cued the subject to initiate the task production within a 5-s period, which was followed by a 2-s period of image acquisition.

Subjects were instructed to listen to an auditory sample of eight different English sentences (e.g., “Jack ate eight apples,” “Tom is in the army”) delivered one at a time by the same female native English speaker through MR-compatible headphones within a 3.6 s period. When cued by an arrow, subjects produced the task (i.e., repeated the sentence once) within a 5 s period, which was followed by a 2 s whole-brain volume acquisition (Figure 1). Rest periods without any auditory input or task production were incorporated as a baseline condition. Each subject completed four functional runs, consisting of 24 task and 16 resting conditions.

### Image Processing

#### Anatomical MRI

Whole-brain T1-weighted images were analyzed using the automated “recon-all” function implemented in FreeSurfer software. Briefly, the processing included motion correction, intensity normalization, skull-stripping, volumetric registration with labeling, tissue segmentation, and gray-white interface and pial surface delineation. Cortical parcellation was performed using the Destrieux atlas, which assigned neuroanatomical labels to each location on the cortical surface while incorporating geometric information derived from the subject’s cortical model (Fischl, 2012). All cortical parcellations were visually inspected for accuracy and, if necessary, corrected manually.

#### Functional MRI

Image analysis was performed using the standard *afni_proc.py* pre-processing pipeline in AFNI software, which included removal of spikes, registration, alignment of the EPI volume to the anatomical scan, spatial normalization to the AFNI standard Talairach-Tournoux space, spatially smoothed with a 4-mm Gaussian filter, scaling of each run mean to 100 for each voxel, and motion scrubbing. A task regressor was convolved with a canonical hemodynamic response function and entered into a multiple regression model to predict the observed BOLD response during speech production. Group analysis was carried out using a two-sided one-sample *t*-test. The statistical threshold was set at a voxel-wise and cluster-wise corrected *p* ≤ 0.001, with minimal cluster size of 100 voxels using AFNI’s 3dClustSim.

#### Cortical Regions-of-Interest

**FIGURE 2:**
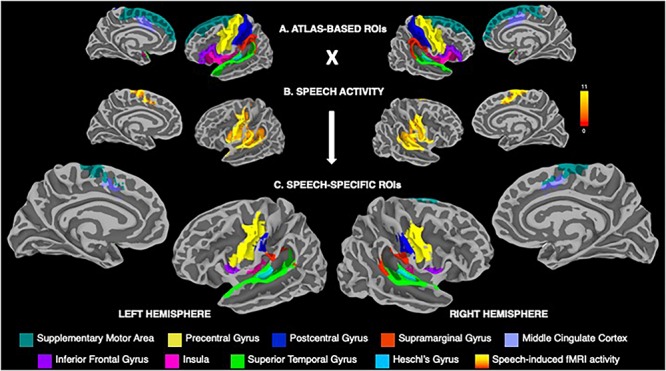
**(A)** Visualization of atlas-based anatomical regions-of-interest (ROIs) within the speech production network based on the Destrieux atlas parcellation, including the precentral, postcentral and inferior frontal gyri, supplementary motor area, middle cingulate cortex, supramarginal, superior temporal and Heschl’s gyri, and the insula. **(B)** Group statistical map of whole-brain activation during speech production across males and females. Color bar represents the *t*-score at *p* ≤ 0.001. **(C)** Speech-specific cortical ROIs derived from conjoining the atlas-based anatomical ROIs with the binarized map of speech-related brain activity. The ROIs are color-coded based on their anatomical affiliation and displayed on the FreeSurfer average template.

Consistent with the previous studies of neural activity during speech production (e.g., Tourville and Guenther, 2003; Simonyan and Fuertinger, 2015; Simonyan et al., 2016; Basilakos et al., 2018; Kearney and Guenther, 2019), the cortical regions-of-interest (ROIs) included the precentral, postcentral and inferior frontal gyri, supplementary motor area, middle cingulate cortex, supramarginal (SMG), superior temporal (STG) and Heschl’s gyri, and insula (Figure 2A). Following the extraction of parcellated Destrieux atlas-based ROIs, a further delineation of these regions included their restriction to areas activate during speech production (Figure 2B). For this, the group mean activity map during speech production was binarized, warped into MNI space using AFNI’s 3dWarp, transformed from the volumetric space to the surface space using AFNI’s *3dVol2Surf* and conjoined with atlas-based ROIs, resulting in speech-specific cortical ROIs (Figure 2C).

In each subject, the mean CT measure was extracted from each speech-specific cortical ROI using Freesurfer’s *mri_segstats.* Multivariate analysis of covariance, accounting for age as a covariate, was used to examine between-group differences in CT measures within each right and left hemisphere. Separately, within-group differences in CT measures between hemispheres were examined using paired *t*-tests. Statistical significance was Bonferroni-corrected by the number of ROIs used in the analysis and set at *p* < 0.005.

## Results

Both males and females exhibited a typical pattern of cortical activity during speech production, which involved primary sensorimotor, premotor, inferior frontal, middle cingulate, auditory, inferior parietal and insular regions (Figure 2A), in agreement with other studies investigating speech production (e.g., Tourville and Guenther, 2003; Fuertinger et al., 2015; Simonyan et al., 2016; Basilakos et al., 2018; Kearney and Guenther, 2019). For further analysis, this activity was restricted to the *a priori* delineated cortical structural ROIs, as outlined above and illustrated in Figure 2.

**FIGURE 3:**
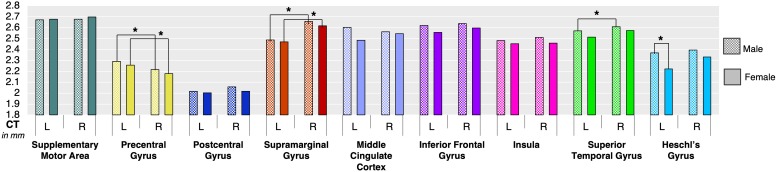
Boxplot shows mean cortical thickness (in mm) and standard error in each speech-specific cortical region-of-interest in males and females. Asterisk (^*^) depicts statistically significant differences between males and females as well as within each male and female group.

Analysis of regional CT showed that females had significantly greater left Heschl’s gyrus compared to males (*p* = 0.002) (Figure 3 and Table 1). None of other cortical regions showed significant differences in CT between the male and female groups (*p* ≥ 0.11).

**TABLE 1:** Mean cortical thickness of speech-related regions in females (♀) and males.(♂)

**Regions-of-interest**	**Mean ± Standard Error CT**				***P***			
	**♀**		**♂**		**Between groups within hemisphere**		**Within groups between hemispheres**	
	**L**	**R**	**L**	**R**	**L♂vs. L♀**	**R♂vs. R♀**	**L♀vs. R♀**	**L♂vs. R♂**
Superior temporal gyrus	2.57 ± 0.03	2.61 ± 0.03	**2.51 ± 0.03**	**2.57 ± 0.03**	0.21	0.44	0.07	**0.004**
Insula	2.48 ± 0.02	2.51 ± 0.03	2.45 ± 0.03	2.46 ± 0.04	0.5	0.34	0.15	0.73
Inferior frontal gyrus	2.62 ± 0.03	2.64 ± 0.03	2.56 ± 0.04	2.60 ± 0.03	0.2	0.44	0.45	0.10
Precentral gyrus	**2.29 ± 0.03**	**2.22 ± 0.03**	**2.26 ± 0.03**	**2.18 ± 0.03**	0.44	0.37	**<0.0001**	**<0.0001**
Postcentral gyrus	2.02 ± 0.03	2.06 ± 0.03	2.00 ± 0.03	2.02 ± 0.03	0.78	0.41	0.05	0.60
Heschl’s gyrus	**2.37 ± 0.03**	2.39 ± 0.03	**2.22 ± 0.03**	2.33 ± 0.04	**0.002**	0.31	0.48	0.02
Supplementary motor area	2.67 ± 0.03	2.68 ± 0.03	2.68 ± 0.04	2.70 ± 0.04	0.84	0.65	0.84	0.53
Supramarginal gyrus	**2.49 ± 0.03**	**2.66 ± 0.03**	**2.47 ± 0.03**	**2.62 ± 0.03**	0.76	0.38	**<0.0001**	**<0.0001**
Middle cingulate cortex	2.60 ± 0.0	2.56 ± 0.03	2.48 ± 0.07	2.54 ± 0.03	0.14	0.76	0.23	0.31

*Statistically significant differences between males and females as well as within each male and female group are shown in bold.*

However, within each group, both females and males exhibited left-hemispheric asymmetry of precentral gyrus (*p* ≤ 0.001) and right-hemispheric asymmetry of SMG (*p* ≤ 0.001). In addition, males showed right-hemispheric asymmetry of STG (*p* = 0.004) (Figure 3 and Table 1).

## Discussion

Our study demonstrated the presence of speech-specific sexual dimorphism in CT of primary auditory cortex within the Heschl’s gyrus. In addition, structural hemispheric asymmetry both in males and females was identified in selected brain regions controlling speech motor execution (precentral gyrus), auditory processing (STG) and sensorimotor integration (SMG).

Auditory cortex within the Heschl’s gyrus is known to encode short-latency temporal features of auditory stimuli that have repetition rates within the range of the fundamental frequency of human voice (Belin et al., 1998; Price, 2000; Zatorre, 2001; Scott and Wise, 2004; Brugge et al., 2008, 2009; Warrier et al., 2009; Chevillet et al., 2011; Nourski and Brugge, 2011; Kusmierek et al., 2012). Distinct functional parcellations of core and non-core auditory areas within the Heschl’s gyrus process natural human vocalizations and pitch perturbations in the auditory feedback (Behroozmand et al., 2016). Earlier lesion studies have demonstrated that damage to the left auditory cortex often results in deficits of temporal processing, manifesting as a speech disorder (Damasio and Damasio, 1980; Phillips and Farmer, 1990). Along these lines, our finding of greater CT in the left Heschl’s gyrus in females than males suggests that structural enhancement of this region might be associated with sex-specific differences in processing of auditory cues during speech production as well as contribute to increased prevalence of speech and language developmental disorders in males (Shriberg and Kwiatkowski, 1994; Law et al., 1998; Keating et al., 2001).

We further found between-hemispheric rightward asymmetry of the STG in males but not females. This finding is in line with earlier studies that suggest the influence of genes involved in steroid hormone receptor activity in this region. Specifically, testosterone and progesterone may exert opposing effects on the STG structural organization by promoting its rightward asymmetry in males and forging its structural symmetry in females, respectively (Geschwind and Galaburda, 2003; Guadalupe et al., 2015). This is consistent with the hypothesis that region-specific sexual dimorphisms might be related to factors affecting *in utero* and early postnatal sexual differentiation of the neural system (Goldstein, 2001).

In both males and females, a characteristic feature of CT organization within the speech production network was its rightward asymmetry of the SMG and leftward asymmetry of the precentral gyrus, encompassing primary motor and premotor cortical areas. The SMG is involved in higher-order processing and plays an important role in the coordination of speech-motor learning, sensorimotor adaptation, phonological decisions, auditory error recognition, and speech onset monitoring (Price et al., 1997; McDermott et al., 2003; Shum et al., 2011; Sliwinska et al., 2012; Deschamps et al., 2014; Kort et al., 2014; Fuertinger et al., 2015). In line with a recent study showing involvement of the right SMG in the prosodic and paralinguistic aspects of speech production (Lindell, 2006), our results suggest that rightward asymmetry of this region may be important for higher-order integration of phonological processing in both males and females. Similarly, leftward CT asymmetry in the precentral gyrus, specifically encompassing its speech motor cortex, may be linked to the general left-hemispheric dominance of this region in the fulfillment of motor tasks in right-handed males and females. This finding also substantiates the left-hemispheric dominance of functional network originating from the laryngeal motor cortex (Lindell, 2006; Simonyan et al., 2009).

Putting the current findings in context with the previous literature, it is important to note that earlier investigations of CT asymmetry have used large atlas-based brain regions that were not confined to speech-related brain activity. This might have led to the mixed reports of both left- and right-hemispheric lateralization of the precentral gyrus and STG in both males and females (Luders et al., 2006; Guadalupe et al., 2015; Kong et al., 2018). Additionally, some studies have reported left-hemispheric asymmetry of CT and regional surface area in the SMG (Lyttelton et al., 2009; Koelkebeck et al., 2014; Plessen et al., 2014; Maingault et al., 2016), while others have found no such differences in this region (Luders et al., 2006; Koelkebeck et al., 2014; Kong et al., 2018). While these inconsistencies might indicate the absence of population-level CT asymmetries (Kong et al., 2018), they may also stem from a failure to account for sex differences in structural organization of the speech production network.

In summary, this study provides evidence for the existence of sex-specific structural dimorphisms within the cortical speech production circuitry. Our findings highlight the importance of the inclusion of sex as a biological variable in research on neural correlates of speech control. Furthermore, our data suggest that examination of speech-specific cortical morphology benefits from restricting analysis to anatomical areas that are functionally active during this complex behavior.

## Data Availability

All datasets generated for this study are included in the manuscript and/or the supplementary files.

## Ethics Statement

This study was carried out in accordance with the recommendations of the Internal Review Board of Massachusetts Eye and Ear Infirmary. All subjects gave written informed consent in accordance with the Declaration of Helsinki. The protocol was approved by the Internal Review Board of Massachusetts Eye and Ear Infirmary.

## Author Contributions

KS collected the data. KS, LdLX, and SH designed the study and statistical methods. KS critically reviewed the manuscript and obtained funding. LdLX and SH analyzed the data and drafted the manuscript.

## Conflict of Interest Statement

The authors declare that the research was conducted in the absence of any commercial or financial relationships that could be construed as a potential conflict of interest.
